# Bile acids drive chemotaxis of *Clonorchis sinensis* juveniles to the bile duct

**DOI:** 10.1371/journal.pntd.0006818

**Published:** 2018-10-01

**Authors:** Shunyu Li, Won Gi Yoo, Jin-Ho Song, Tae Im Kim, Sung-Jong Hong

**Affiliations:** 1 Department of Medical Environmental Biology, Chung-Ang University College of Medicine, Seoul, Republic of Korea; 2 Department of Pharmacology, Chung-Ang University College of Medicine, Seoul, Republic of Korea; 3 Division of Planning and Management, Nakdong-gang National Institute of Biological Resources, Sangju-si, Gyeongbuk, Republic of Korea; Queen's University Belfast, UNITED KINGDOM

## Abstract

Clonorchiasis is a neglected tropical disease caused by Chinese liver fluke, *Clonorchis sinensis* infection. *C*. *sinensis* is a biological carcinogen causing cholangiocarcinoma in humans. In the mammalian host, *C*. *sinensis* newly excysted juveniles (CsNEJs) migrate from the duodenum into the bile duct. Bile drives the chemotactic behavior of CsNEJs. Little is known about which components of bile induce the chemotaxis. We designed a chemotaxis assay panel and measured the chemotactic behavior of CsNEJs in response to bile or bile acids. The CsNEJs migrated toward 0.1–1% bile but away from 5–10% bile. The CsNEJs showed strong chemoattraction to cholic acid ≥25 mM, but chemorepulsion to lithocholic acid ≥0.25 mM. To the CsNEJs, mixture of cholic acid and lithocholic acid was chemoattractive at a ratio greater than 25:1 but chemorepulsive at one smaller than that. Regarding migration in the mammalian hosts, high concentration of lithocholic acid in the gallbladder bile may repel CsNEJs from entering it. However, bile in the hepatic bile duct has a chemoattractive strength of cholic acid but a trace amount of lithocholic acid. Collectively, our results explain why the CsNEJs migrate principally to the hepatic bile ducts, bypassing the gallbladder.

## Author summary

We previously reported that *Clonorchis sinensis* newly excysted juveniles (CsNEJs) were chemotactically attracted to bile. However, there is still a paucity of information regarding which components and what concentration of bile induce the chemotactic behavior. Here, we show, among various bile components tested, two have opposing chemotactic influences on the CsNEJs; cholic acid was characterized as a chemoattractant and lithocholic acid as a chemorepellent. Chemorepulsive migration was dependent on the concentration of lithocholic acid. Notably, the ratio (25:1) of cholic acid and lithocholic acid plays a critical role in defining chemotactic preferences of CsNEJs. We suspect that this bile acid ratio directs the parasites in the mammalian host, i.e. the high concentration of lithocholic acid in the gallbladder bile may repel CsNEJs from entering it. Bile in the hepatic bile duct has a chemoattractive level of cholic acid but a trace amount of lithocholic acid. These findings may explain why the CsNEJs preferentially migrate to the common and hepatic bile ducts rather than the gallbladder. Deeper understanding on the parasitism of the liver fluke is likely to have major implications for the studies on other parasites.

## Introduction

Many parasites seek out and invade hosts using host-emitted chemical cues, exhibiting a trait known as chemotaxis. Recent studies have encompassed a range of examples, including the miracidia of *Schistosoma* species swim along a chemical gradient toward the snail host [1, 2]. The larvae of *Echinostoma* species, both miracidia and cercariae, locate their snail hosts by sensing chemotactic cues [3]. Invasion by *S*. *mansoni* schistosomula is induced by chemo-orientation toward d-glucose and l-arginine in the host serum [4]. When *Diplostomum spathaceum* cercariae invade fish, they recognize monosaccharides, glycoproteins, and fatty acids [5]. Within the hosts, these kinds of chemotaxis also are crucial for successful parasitism and survival although the parasites can migrate to atypical location [6].

Bile and bile acids provide pivotal cues to gastrointestinal parasites. Glycine-conjugated cholic acid stimulates excystation of *Fasciola hepatica* metacercariae [7]. Whole bovine bile and dehydrocholic acid stimulate the locomotor cycle of *F*. *hepatica* juveniles [8]. Oviposition of *S*. *mansoni* adults is increased by bile components, especially tauroursodeoxycholic acid [9]. Survival rate of newly excysted *Clonorchis sinensis* juveniles (CsNEJs) increases in low concentration of bile [10]. Bile acids and conjugated bile salts, except lithocholic acid, enhance activity of CsNEJs [10].

Bile acids, which comprise most of the organic compounds in bile, include cholic acid (34%), chenodeoxycholic acid (39%), and deoxycholic acid (26%) as major components and lithocholic acid (<0.5%) as a trace constituent in both gallbladder and hepatic bile duct [11, 12]. In mammals, the main function of bile acids is to facilitate the formation of micelles for fat absorption. Several bile acids, such as whole bovine bile, dehydrocholic acid and tauroursodeoxycholic acid, are known to influence physiological or kinetic activities of flukes [8–10]. On the basis of these studies, we suspected that the chemotactic behavior of CsNEJs toward bile could be associated with bile acids.

As a bile-dwelling parasite, *C*. *sinensis* is the most prevalent liver fluke in East Asian countries, infecting more than 200 million people [13]. The World Health Organization has recognized *C*. *sinensis* as a biological carcinogen for cholangiocarcinoma in human [14]. The mammalian hosts become infected by eating freshwater fish containing *C*. *sinensis* metacercariae. The ingested metacercariae excyst in the duodenum, and CsNEJs promptly migrate to the intrahepatic bile duct. Bile is assumed to be a chemoattractant to CsNEJs, since they migrate only to the bile duct of a rabbit whose gallbladder is stimulated to release bile [6]. However, it is not known which component of bile drives the migration of CsNEJs, and why they prefer to move to the liver rather than to enter the proximal and bile-rich gallbladder.

In order to investigate chemotactic migration of *C*. *sinensis*, we designed and fabricated a custom-made chemotaxis assay trough similar to the tubular route from the ampulla of Vater to the biliary passages in the mammalian host. With the chemotaxis assay panel, we investigated which bile components induce this peculiar chemotactic behavior of CsNEJs.

## Materials and methods

### Preparation of newly excysted *C*. *sinensis* juveniles

Naturally infected *Pseudorasbora parva*, the second intermediate host of *C*. *sinensis*, was purchased at Hunhe fish market in Shenyang, Liaoning Province, People’s Republic of China. The fish was ground and digested in artificial gastric juice (0.5% pepsin [MP Biochemicals Co., Solon, OH, USA], pH 2.0) for 2 h at 37°C [15]. The solid matter was removed from digested content by filtration through a sieve of 212-μm mesh diameter. The *C*. *sinensis* metacercariae were collected using sieves of 106- and 53-μm mesh diameter and washed thoroughly several times with 0.85% saline. The *C*. *sinensis* metacercariae were gathered under a dissecting microscope and stored in phosphate-buffered saline (PBS) at 4°C until use. The metacercariae were excysted in 0.005% trypsin (Difco, Detroit, MI, USA) and used as CsNEJs for experiments.

### Customized chemotaxis assay panel

**Fig 1:**
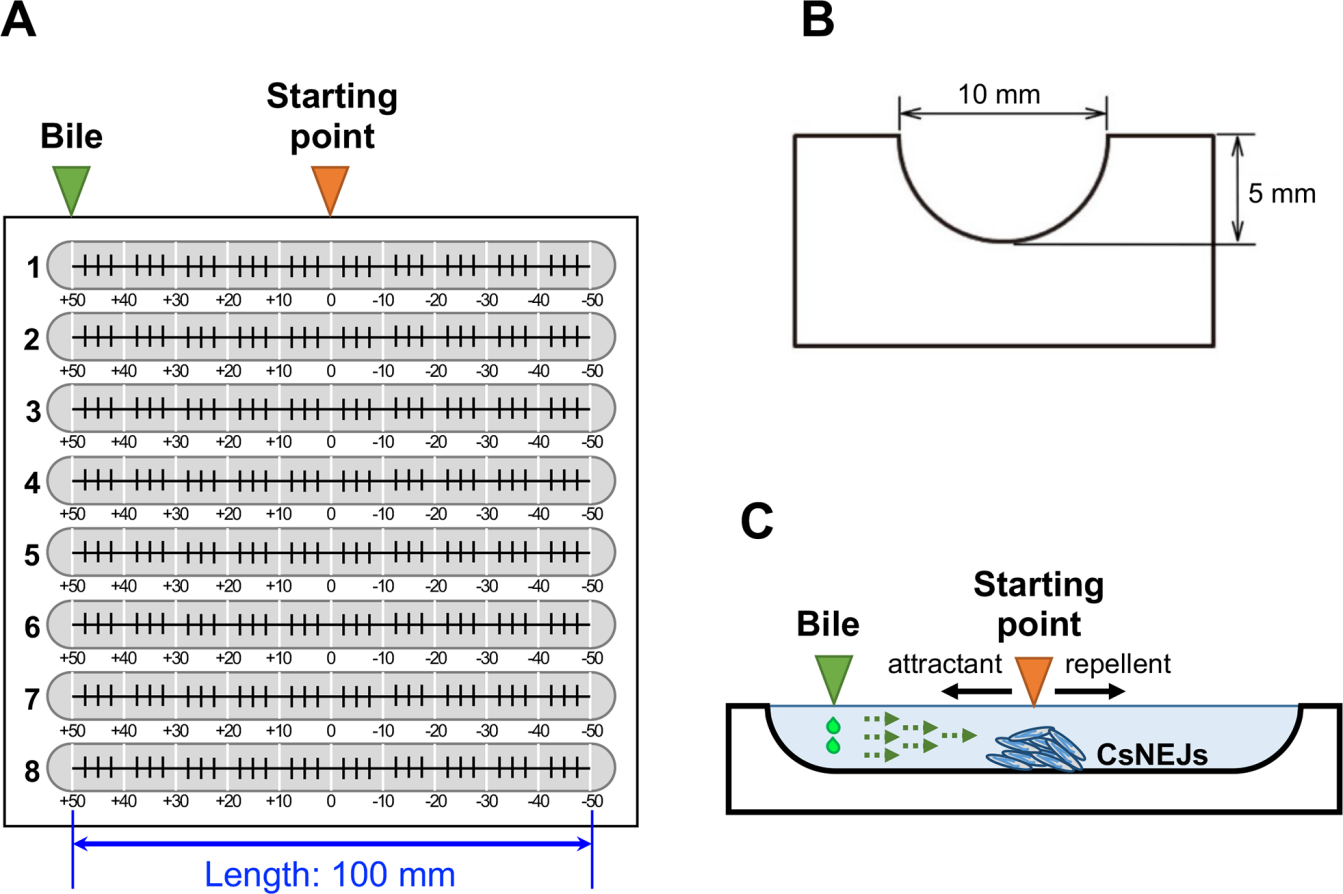
The custom-made chemotaxis assay panel. (**A**) The chemotaxis assay panel with 8 troughs. The troughs were carved in a polycarbonate block. Each 100 mm-long trough was graduated with a centimeter scale (*white lines*), and with a finer scale (*black teeth*) on the bottom surface. (**B**) Cross section of the trough. (**C**) Experimental conditions in the trough. The *rightward broken arrows* represent diffusion of the bile solutions. Migration distance was measured to evaluate whether a bile acid was chemotactically attractive (*leftward solid arrow*) or repellent (*rightward solid arrow*). The worms were initially placed at the center of the trough (*orange triangle*) and allowed free to respond to bile or bile acid solutions dropped at the left end (*green triangle*).

A custom-made chemotaxis assay panel with 8 troughs was crafted. Eight half-round troughs with dimensions of 100 mm long, 10 mm wide and 5 mm deep, were carved in a polycarbonate block (Fig 1A and 1B). Each trough was graduated, with 0 at the center, +10 to +50 mm on the left side, and −10 to −50 mm on the right side. CsNEJs were placed at the center of trough in the custom-made chemotaxis assay panel, and then serially diluted bile solutions were dropped at one end (Fig 1A and 1C).

### Chemotactic assays in walk-in incubator

All experiments were performed at our lab in Chung-Ang University College of Medicine. A walk-in incubator (Model No. J-RHC; JISICO CO., LTD, Seoul, Korea; http://www.jisico.co.kr) was built-in to maintain constant temperature and humidity with dimensions, 250 cm wide, 296 cm long and 210 cm high (S1A and S1B Fig). For all experiments, the walk-in incubator was equilibrated at temperature 37°C and at humidity 80% for 1 h prior to the experiments.

### Assay of chemotactic migration

In all chemotaxis assays, 1× Locke’s solution was used as a base solution [10]. Each trough was filled with 1 ml of 1× Locke’s solution and approximately 20 CsNEJs were placed at the center 0 point using a micropipette. After allowing 5 min for adaptation, CsNEJs were stimulated by dropping bile or bile acid solutions (Sigma-Aldrich, St. Louis, MO, USA) at the +50 mm point. Behavior and migration distance of CsNEJs were observed under a dissecting microscope at each time point. All experiments were performed inside a walk-in incubator maintained at 37°C and 80% humidity as described above. To minimize temperature fluctuation, the chemotaxis panel was covered with acrylic lid except when the chemicals were applied or CsNEJs were observed.

Bile or bile acid solutions were freshly made immediately before use. For bile chemotaxis assay, 10 μl of 0.01–10% bile solution was dropped at the +50 mm point, and then migration distance of CsNEJs was recorded at a given time interval. As a control, 10 μl of 1× Locke’s solution was used. Four kinds of bile acids, i.e., cholic acid, deoxycholic acid, chenodeoxycholic acid and lithocholic acid, were used in the evaluation. Four microliters of bile acid solution were dropped at the +50 mm point in the trough, and then migration distance of CsNEJs was recorded. As bile acids were dissolved in dimethyl sulfoxide (DMSO, Sigma-Aldrich, St. Louis, MO, USA), 4 μl DMSO was dropped at the +50 mm point as a control. The CsNEJs that died during the assay were excluded.

### Repetitive cholic acid stimulation

To allow a longer migration distance, the scale in the chemotaxis assay trough was rearranged: the starting 0 point was moved to the right end of the trough and the graduation was marked +10–+80 mm toward the left end. At the beginning, CsNEJs were placed at 0 point in the trough and 4 μl of 50 mM cholic acid was dropped at the +30 mm. Every 10 min, the cholic acid solution was sequentially dropped at the + 50 mm and +70 mm point in the trough.

### Data analysis

Mean chemotactic distance (mm) was calculated by summing the migration distances of all CsNEJs and dividing it by the number of CsNEJs. All experiments were performed in triplicate with different batches of CsNEJs. Each value was presented as a mean ± standard error of mean. The significance of difference was statistically analyzed by Student’s *t*-test with *p*-value less than 0.05 considered significant.

## Results

### Chemotactic responses to bile

**Fig 2:**
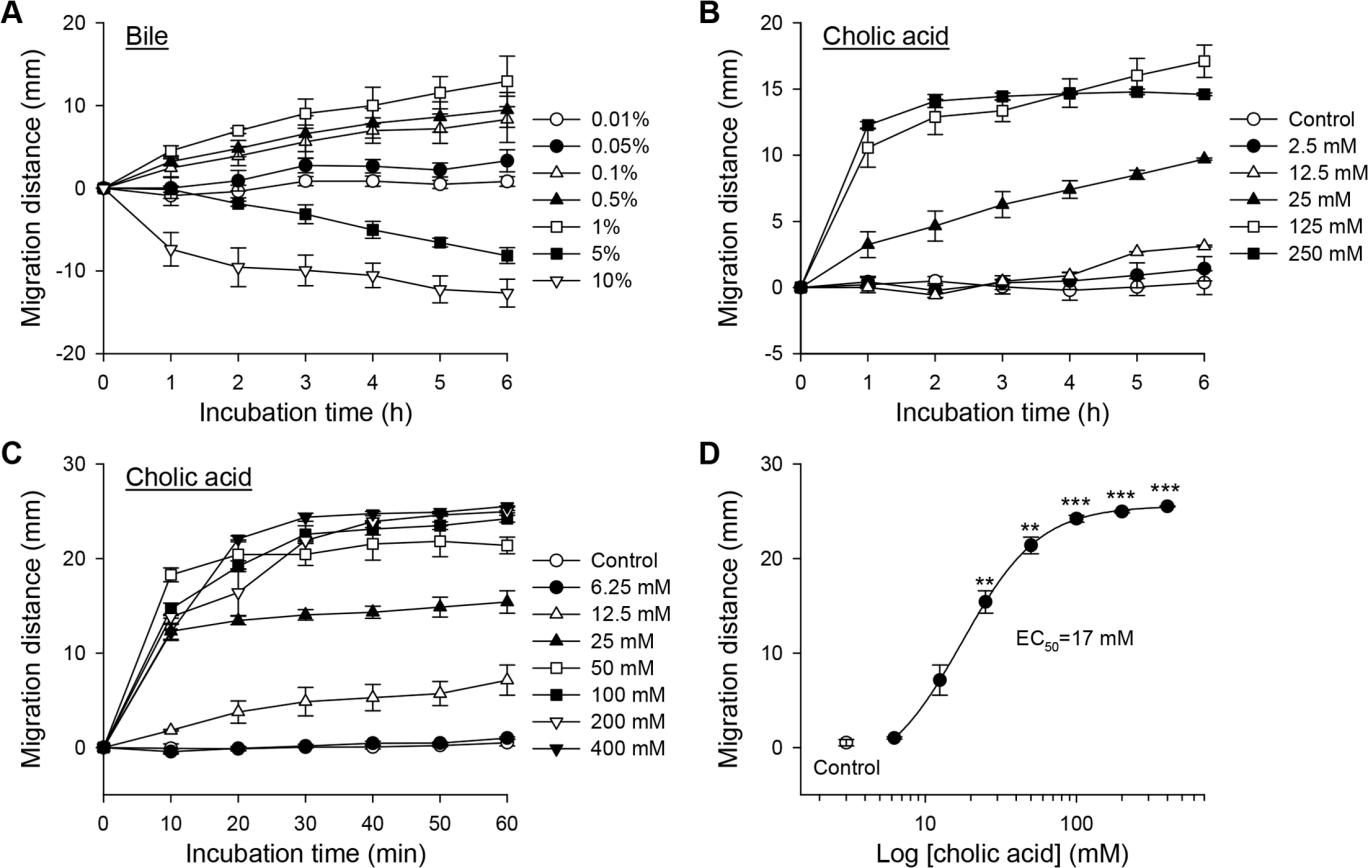
Chemotactic response of newly excysted *Clonorchis sinensis* juveniles (CsNEJs) to bile and cholic acid. (**A–C**) Chemotactic migration of CsNEJs in response to various concentrations of bile for 6 h (**A**), and cholic acid for 6 h (**B**) and 60 min (**C**). (**D**) Concentration-response relationship curve of cholic acid at 60 min. Each point represents mean ± standard error of mean. **p<0.01 and ***p<0.001 compared to control.

Chemotactic migration distance of CsNEJs was normalized to the control group (Fig 2A). The CsNEJs responded immediately and migrated concentration-dependently toward 0.1–1% bile, but away from 5 and 10% bile during 6 h of observation. When exposed to 10% bile solution, some CsNEJs shrank, moved very slowly, or stopped moving, but other CsNEJs immediately moved away from the bile solution.

### Cholic acid was a chemoattractant

Chemotactic response of CsNEJs to individual bile acids was investigated. CsNEJs migrated toward cholic acid of concentrations 25 mM or higher during 6 h (Fig 2B). In fact, the majority of the CsNEJs movement was observed during the first 1 h. Closer observation revealed that the CsNEJs migrated very quickly toward cholic acid within as short as 10 min, slowed for 20 min, and then moved only minimally from that point (Fig 2C). The half-maximum effective concentration (EC_50_) was estimated as 17 mM (Fig 2D). The chemoattractive effect of cholic acid was saturated at concentrations above 50 mM. Cholic acid of 25 mM concentration was attractive to CsNEJs at 25–40°C. A higher temperature of 34–40°C induced CsNEJs to migrate a longer distance toward cholic acid than a lower temperature of 25–31°C did (S2 Fig). Deoxycholic acid and chenodeoxycholic acid, major bile components, were less attractive to CsNEJs. Toward the two bile acids, CsNEJs showed insignificant chemotactic behaviors (S3A and S3B Fig).

### Concentration gradient of cholic acid was more attractive

**Fig 3:**
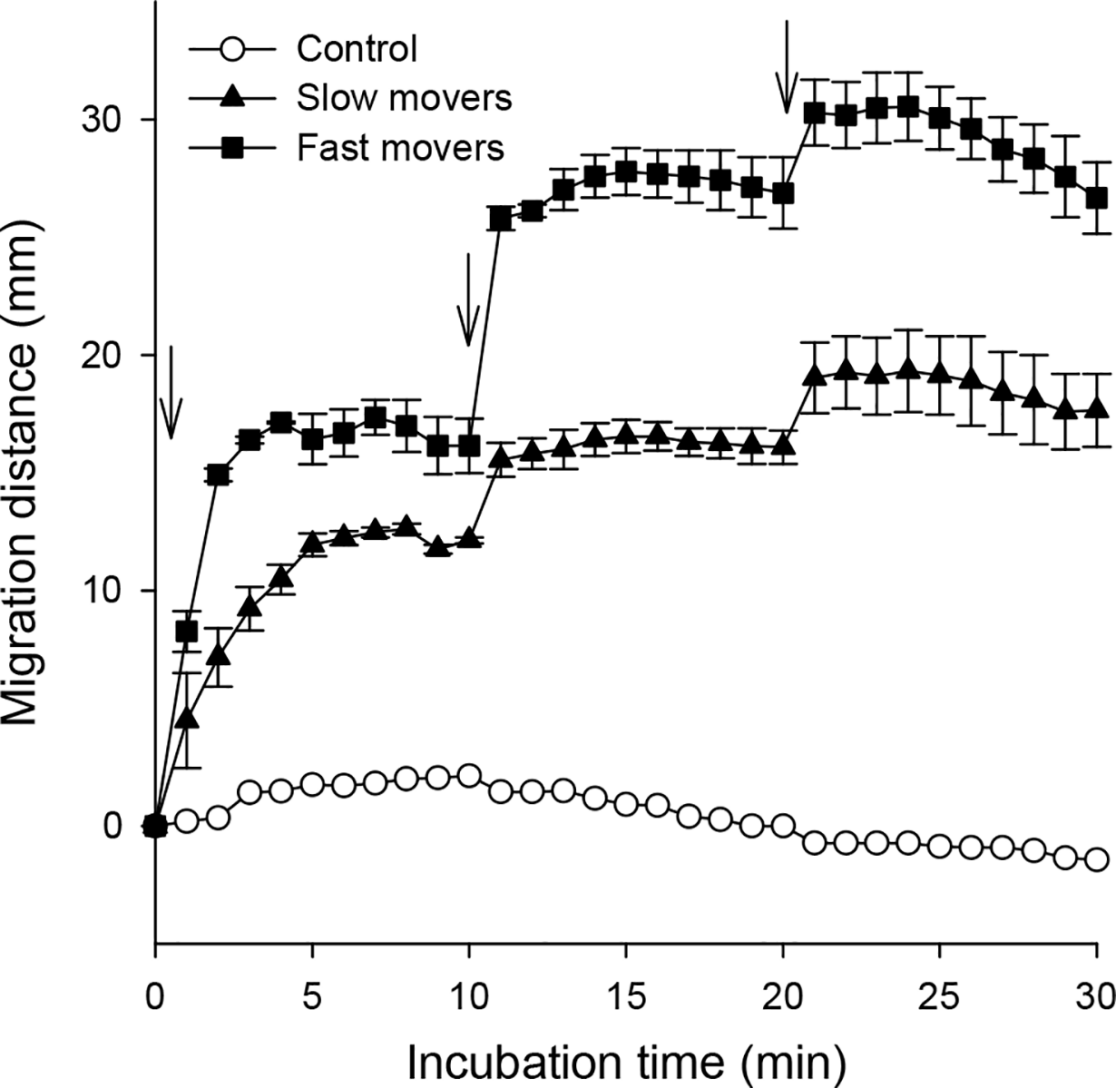
Reactivation of CsNEJs by additional cholic acid. Cholic acid (50 mM; arrow) was applied at 0, 10, and 20 min as indicated with three downward arrows, and migration distance of CsNEJs was measured every min. CsNEJs were arbitrarily grouped into fast (*closed squares*) and slow (*closed triangles*) movers, and they were compared with control group (*open circles*) without cholic acid. Each point represents mean ± standard error of mean.

In the chemotactic assay trough, CsNEJs migrated quickly toward cholic acid for a short time and then lingered at a point. It was suspected that CsNEJs could not sense a concentration gradient of cholic acid because it became dissipated and equilibrated as time passed. To test whether CsNEJs remained sensitive to cholic acid when they stopped moving, they were re-attracted by adding 50 mM cholic acid to the trough. The CsNEJs responded immediately and moved to the added cholic acid, stopped moving within 5 min, and remained at that point. The CsNEJs were found to move at different speeds and were divided arbitrarily into two groups: fast and slow movers. The fast movers reacted swiftly to cholic acid, moved quickly, and migrated farther than the slow movers. Nevertheless, in both groups, the resting CsNEJs still retained sensitivity to cholic acid, and resumed migration to repetitive attractions of cholic acid (Fig 3).

### Lithocholic acid was a chemorepellent

**Fig 4:**
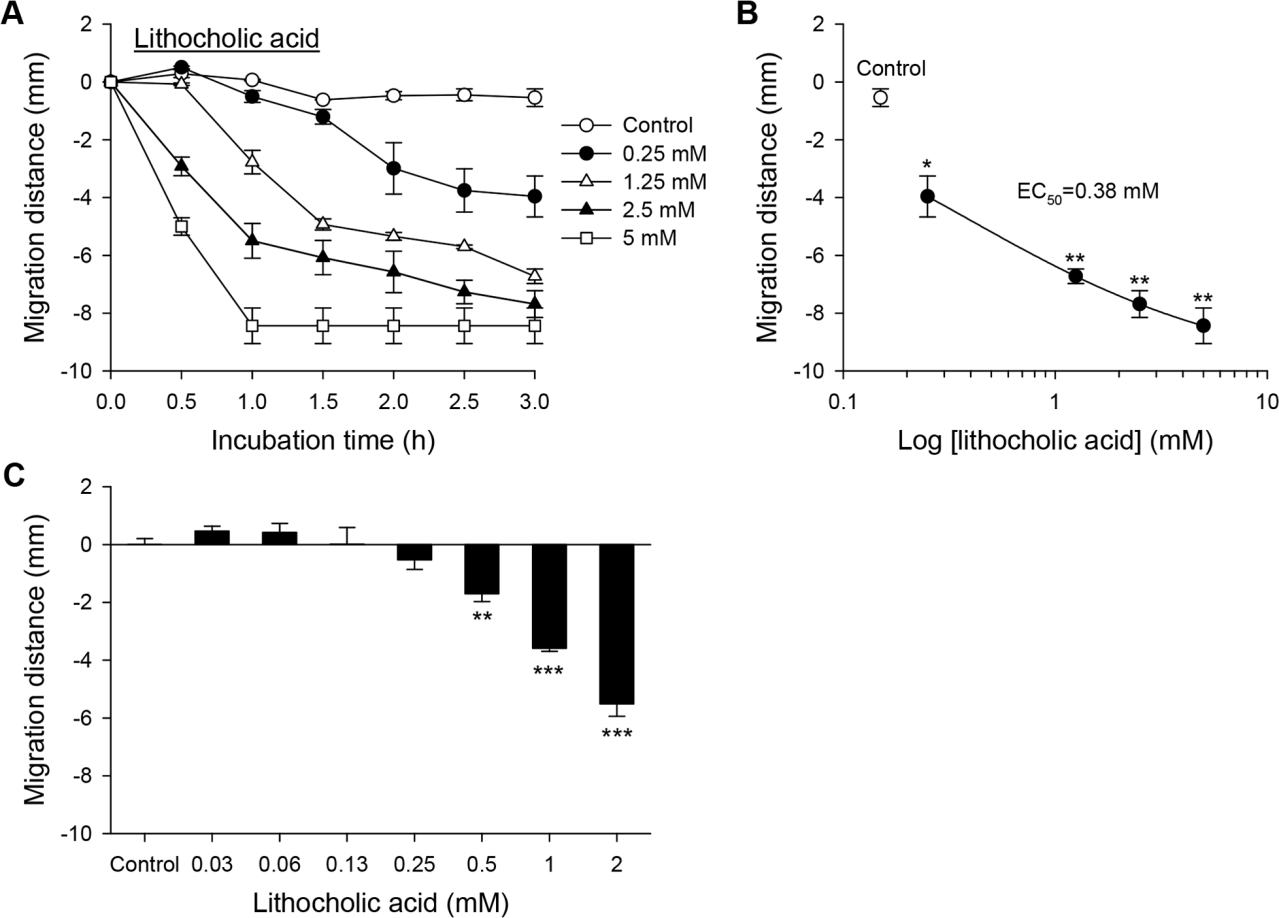
Chemorepellent response of CsNEJs to lithocholic acid. (**A**) Chemorepulsive migration of CsNEJs away from lithocholic acid for 3 h. (**B**) Concentration-response relationship curve of lithocholic acid at 3 h. (**C**) Migration of CsNEJs in response to low concentrations of lithocholic acid for 1 h. Each point represents mean ± standard error of mean. *p<0.05, **p<0.01 and ***p<0.001 compared to control.

In contrast to the chemoattractive cholic acid, lithocholic acid acted as a chemorepellent to CsNEJs. Toward 0.13 mM or lower concentrations of lithocholic acid, CsNEJs wriggled near the starting line, but moved away from lithocholic acid at a concentration of 0.25 mM or higher (Fig 4A and 4C). At a threshold concentration of 0.25 mM lithocholic acid, CsNEJs moved minimally compared to controls for 1.5 h, then slowly migrated 4.0 mm after a total of 3 h. The EC_50_ was estimated as 0.38 mM (Fig 4B). Lithocholic acid at 1.25 mM stimulated CsNEJs to migrate 2.8 mm in 1 h. As the lithocholic acid concentration increased, the migrating speed increased in a concentration-dependent manner with a maximum reaching 8.4 mm at 5 mM. However, as the concentration of lithocholic acid and the duration increased, increasing number of flukes began to shrink and eventually died. When 5 mM lithocholic acid was applied, all CsNEJs died in 1 h (Fig 4A).

### Cholic acid and lithocholic acid guide intrabiliary migration

**Fig 5:**
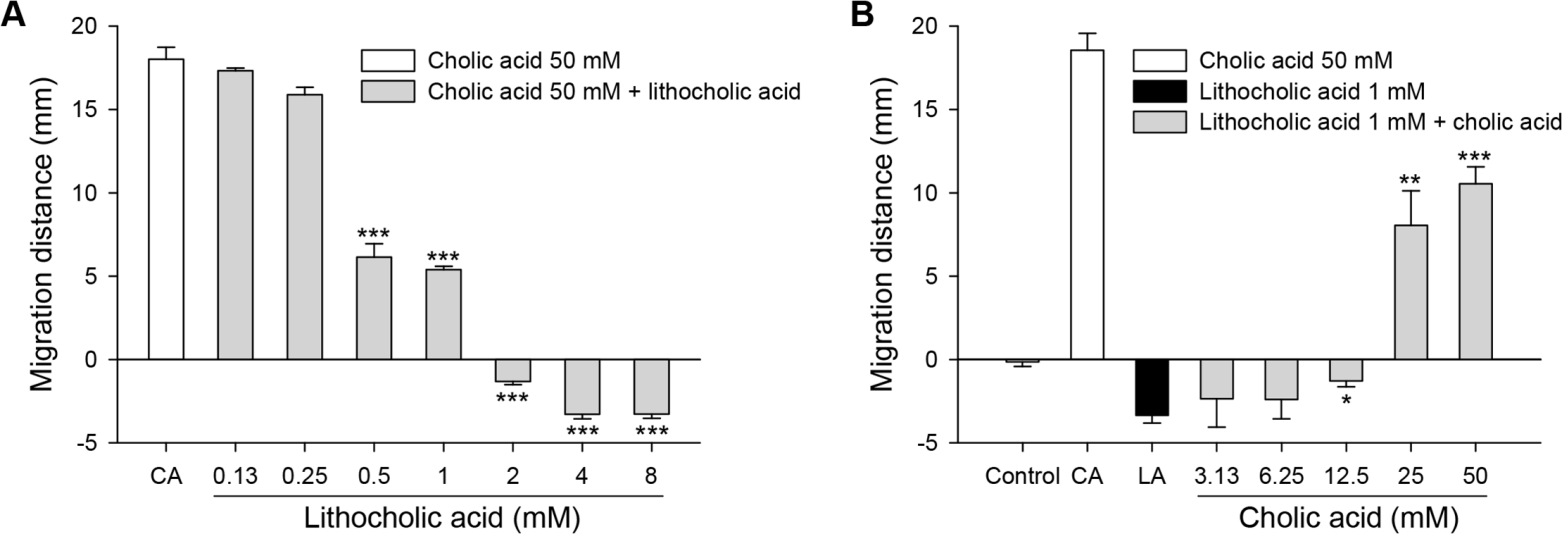
Chemotactic responses to a mixture of cholic acid and lithocholic acid. Chemotactic migration of CsNEJs to a mixed solution of cholic acid and lithocholic acid was examined in 1 h. The mixed solution was made of 50 mM cholic acid and lithocholic acid at concentrations of 0.13–8 mM (**A**), or 1 mM lithocholic acid and cholic acid at concentrations of 3.13–50 mM (**B**). Each point represents mean ± standard error of mean. *p<0.05, **p<0.01 and ***p<0.001 compared to cholic acid only (**A**) or lithocholic acid only (**B**).

CsNEJs encounter chemoattractive cholic acid and chemorepellent lithocholic acid concurrently in the mammalian host. The chemotactic response of CsNEJs to a mixture of cholic acid and lithocholic acid was tested. To a mixed solution with cholic acid fixed at 50 mM, the migration distance decreased as lithocholic acid concentration increased to 1 mM. When lithocholic acid concentration was 2 mM or higher, CsNEJs turned around and moved in the opposite direction from the mixed solution (Fig 5A). Conversely, when lithocholic acid was fixed at 1 mM concentration and cholic acid concentration was successively increased from 3.13 mM, CsNEJs moved away from the mixed solutions of a cholic acid concentration lower than 25 mM, but reversed their migration toward the mixed solution of cholic acid concentration equal to or exceeding 25 mM (Fig 5B). The mixed solution of cholic acid and lithocholic acid was chemoattractive to CsNEJs at a ratio greater than 25:1.

## Discussion

*C*. *sinensis* is a carcinogenic liver fluke thriving in the biliary duct of the final hosts. Bile and bile acids play critical roles in providing physiological stimuli and chemotactic effects on *C*. *sinensis* newly excysted juveniles (CsNEJs) [6, 10]. It was reported that CsNEJs migrated into the hepatic bile duct with bile chemotaxis in the rabbits [6]. However, there has been no report on what components of bile and the bile acids drive the chemotactic migration.

In order to measure and analyze *in vitro* bile chemotaxis of CsNEJs, suitable device and experimental condition is crucial. A major characteristic of the apparatus used for chemotactic analyses was its shape. The plate-based methods were widely used to examine chemotactic responses of *Caenorhabditis elegans* and *Brugia pahangi* [16–20]. Despite the convenience and simplicity of the plate-based method, a variety of special chambers have been developed. In blood flukes, plexiglas choice-chambers, including two-arm-chamber, T-chamber and one-arm-chamber, were previously designed for observing miracidial behavior in a chemical gradient [21]. Circular and T-shape chambers for cercariae and W-shape chamber for schistosomula were applied by others to investigate the worms’ chemotactic response to amino acids [22, 23]. Here, we designed a chemotaxis assay trough similar to the CsNEJ’s migrating route from the duodenum through ampulla of Vater to the biliary passages in the final hosts.

An agarose plate was applied to establish a concentration gradient of a designated solution [5, 20, 23, 24]. It was difficult, however, to make concentration gradient of test solutions and to maintain migration surface with agarose [25]. In the present study, polycarbonate block, not absorbing organic solutes, was employed to make the troughs and to produce natural diffusion of bile and bile acids in the Locke’s solution. To minimize a well-to-well bias, the multiple-arrayed troughs were employed in a plate and two groups of experiments were done simultaneously on one plate.

Although bile is recognized as a considerably toxic even at normal conditions [26], it appeared to have not only toxic but also attractant components to CsNEJs [10]. CsNEJs showed chemoattractive migration toward low concentration of 0.1–1% bile while they revealed chemorepulsive migration away from high concentration of 5–10% bile. Both the chemoattractive and chemorepellent responses were concentration-dependent. This behavior might be related to the decreased survival rate of CsNEJs in a high concentration of bile [10].

CsNEJs were strongly attracted to cholic acid at 25 mM or higher concentration and at temperatures 34–40°C similar to the body temperature of mammalian hosts. Notably, CsNEJs moved quickly toward cholic acid within as short as 10 min and then stayed at the position, probably since the concentration gradient of cholic acid was reduced and equilibrated as time elapsed. The CsNEJs, nonetheless, resumed migration immediately when additional cholic acids were applied. Thus, we theorize in the mammalian final hosts, CsNEJs keep perceiving a concentration gradient of cholic acid flowing down from the ampulla of Vater and migrate up the slippery duodenal surface into the common bile duct.

Upon application of cholic acid, CsNEJs were found to move at different speeds during 30 min of observation and were divided arbitrarily into two groups: fast and slow movers. This finding was in agreement with a previous study in the rabbits [6]. Some CsNEJs migrated quickly from the duodenum, and reached the intrahepatic bile ducts in 7–9 min after inoculation, while other CsNEJs kept moving toward the bile ducts over a prolonged duration [6]. The increased CsNEJs arriving later at the bile duct could be attributed to the slow responders to cholic acid *in vitro* observed in the present study.

Lithocholic acid induced CsNEJs to migrate chemorepulsively away for 1 h at concentrations 1.25 mM or higher with increasing speed in a concentration-dependent manner. The toxicity of lithocholic acid could explain why CsNEJs avoid the concentrated bile. The repulsion is not surprising as lithocholic acid is so hydrophobic and toxic that it causes intrinsic injury to the bile duct of the mice [27]. The chemotactic responses of CsNEJs reflect that the activity and survival of CsNEJs were enhanced in cholic acid media, while they shrank and died in lithocholic acid media [10]. It is therefore proposed that cholic acid is a principal chemoattractive component for CsNEJs in the bile while lithocholic acid acts as a chemorepellent.

In the mammalian host, *C*. *sinensis* adults inhabit the intrahepatic bile duct, but are rarely found in the gallbladder. We suggest that habitat selection by *C*. *sinensis* is associated with avoidance of lithocholic acid. First, bile is secreted from the liver and then held and concentrated in the gallbladder. Bile in the gallbladder is 5–6-fold more concentrated than that in the bile duct [12]. Stronger concentration of lithocholic acid in bile, could hamper the migration of CsNEJs into the gallbladder. Second, CsNEJs revealed chemotactic preference to the mixed bile acids having a ratio of cholic acid to lithocholic acid higher than 25:1. When CsNEJs pass the gallbladder opening in the common bile duct, the biliary bile having much greater ratio of cholic and lithocholic acids could drive CsNEJs to keep migrating to the intrahepatic bile duct.

### Conclusion

Taken together, we found that CsNEJs had chemotaxis to bile, principally to cholic acid. In the mammalian host, upon excystation in the upper duodenum, CsNEJs sense the cholic acid and migrate chemotactically on the duodenal mucosal surface and enter the common bile duct. When encountered *en route* the concentrated gallbladder bile with strong chemorepellent lithocholic acid, the CsNEJs may swerve from it and find their way to the intrahepatic bile duct.

## Supporting information

S1 Fig Front and three-dimensional views of the specially developed walk-in incubator. (**A**) A photo of the front view. (**B**) Schematic presentation of the incubator.
(TIF)

S2 Fig Effect of temperature on chemotaxis of CsNEJs toward cholic acid. Chemotactic migration of CsNEJs toward cholic acid (6.25–400 mM) was measured at various temperatures (25–40°C) for 1 h.
(TIF)

S3 Fig Chemotactic response of CsNEJs to deoxycholic acid and chenodeoxycholic acid. Chemotactic movement of CsNEJs was observed for 5 h in response to various concentrations of deoxycholic acid (**A**) and chenodeoxycholic acid (**B**). Each point represents mean ± standard error of mean.
(TIF)
