# Supplementary figures and images for: Bile acids drive chemotaxis of Clonorchis sinensis juveniles to the bile duct
Source: PLoS Negl Trop Dis. 2018 Oct 1;12(10):e0006818. doi: 10.1371/journal.pntd.0006818 (PMC6181427; doi:10.1371/journal.pntd.0006818)

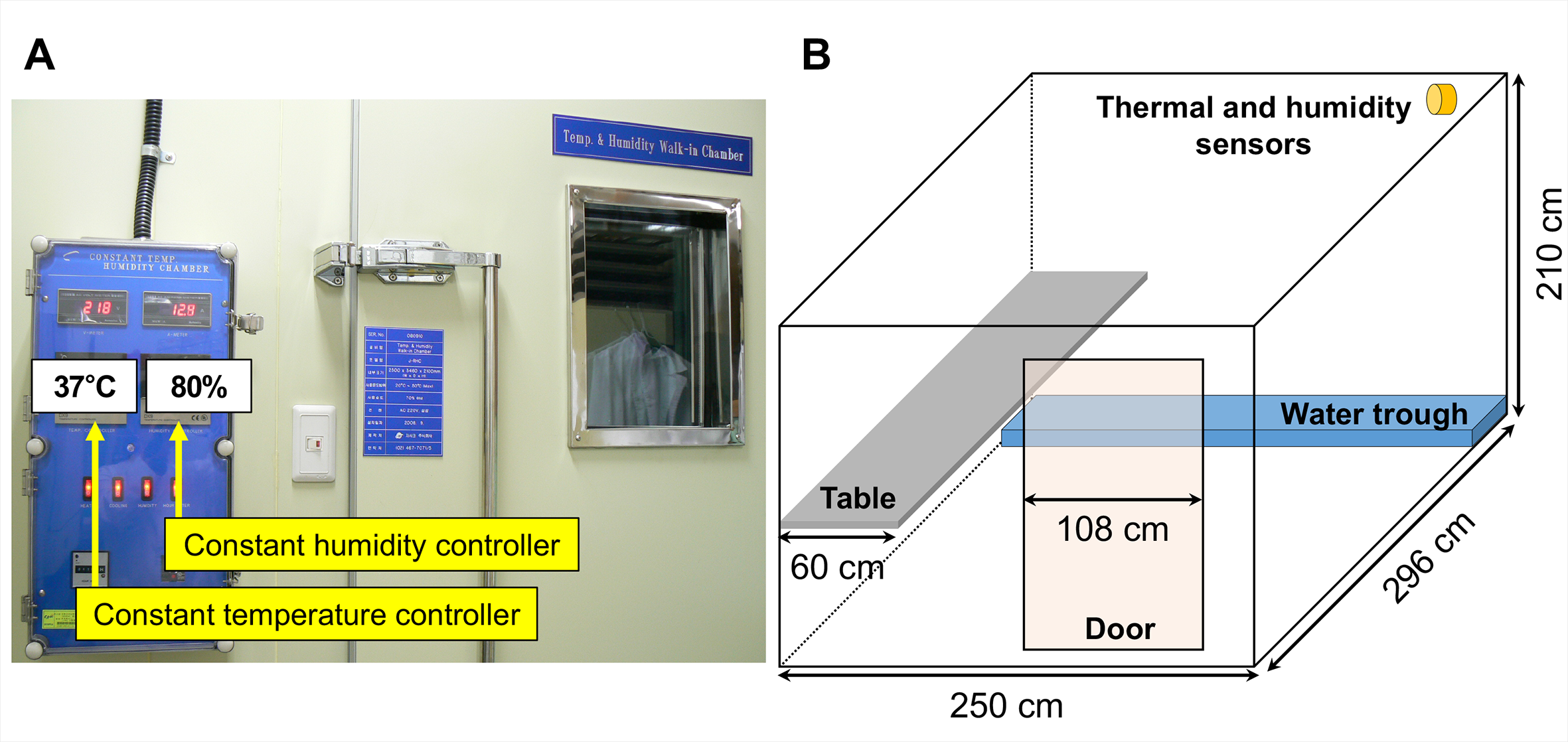

Supplement: S1 Fig — (A) A photo of the front view. (B) Schematic presentation of the incubator. (TIF) [file pntd.0006818.s001.tif]

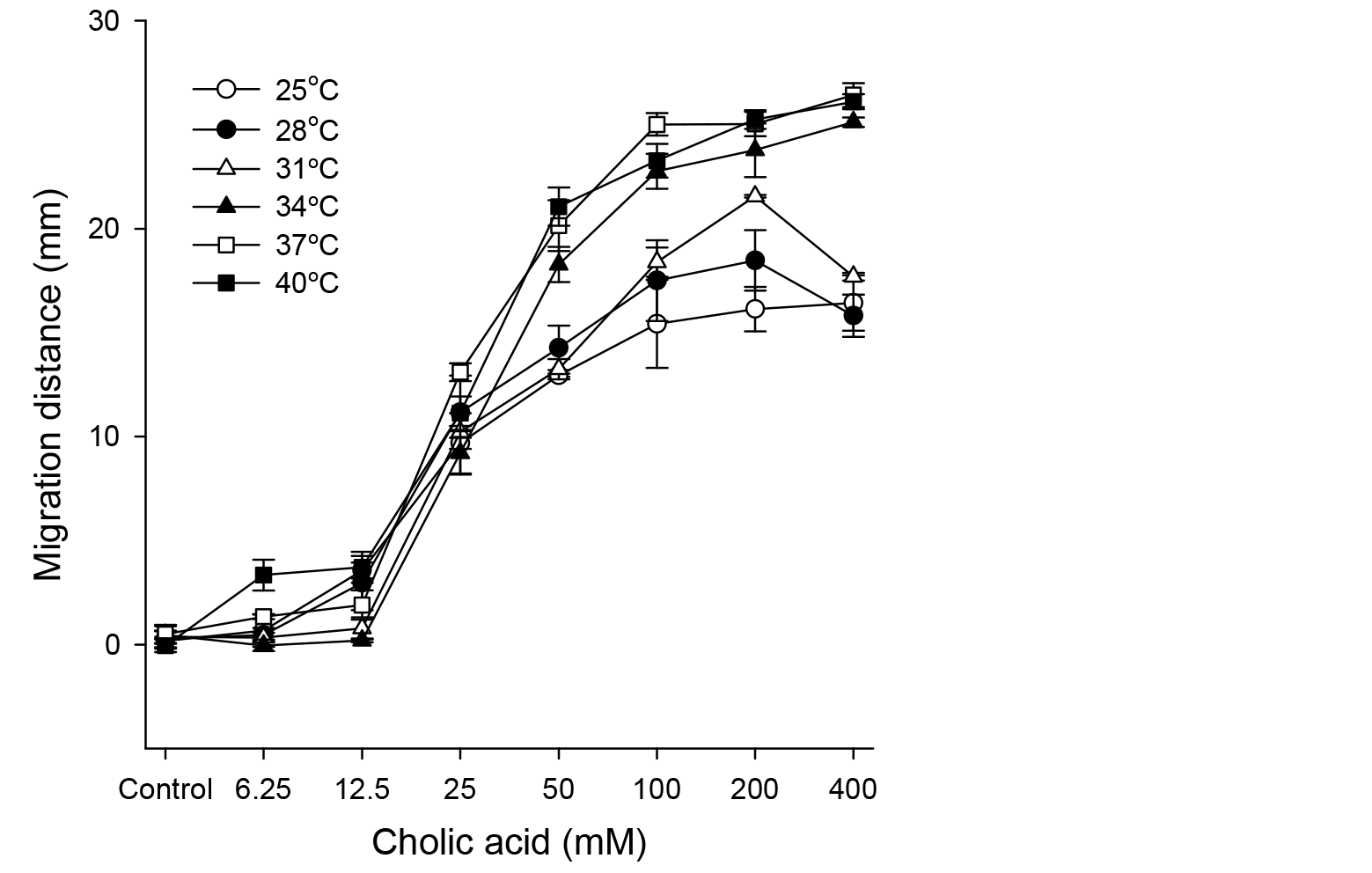

Supplement: S2 Fig — Chemotactic migration of CsNEJs toward cholic acid (6.25–400 mM) was measured at various temperatures (25–40°C) for 1 h. (TIF) [file pntd.0006818.s002.tif]

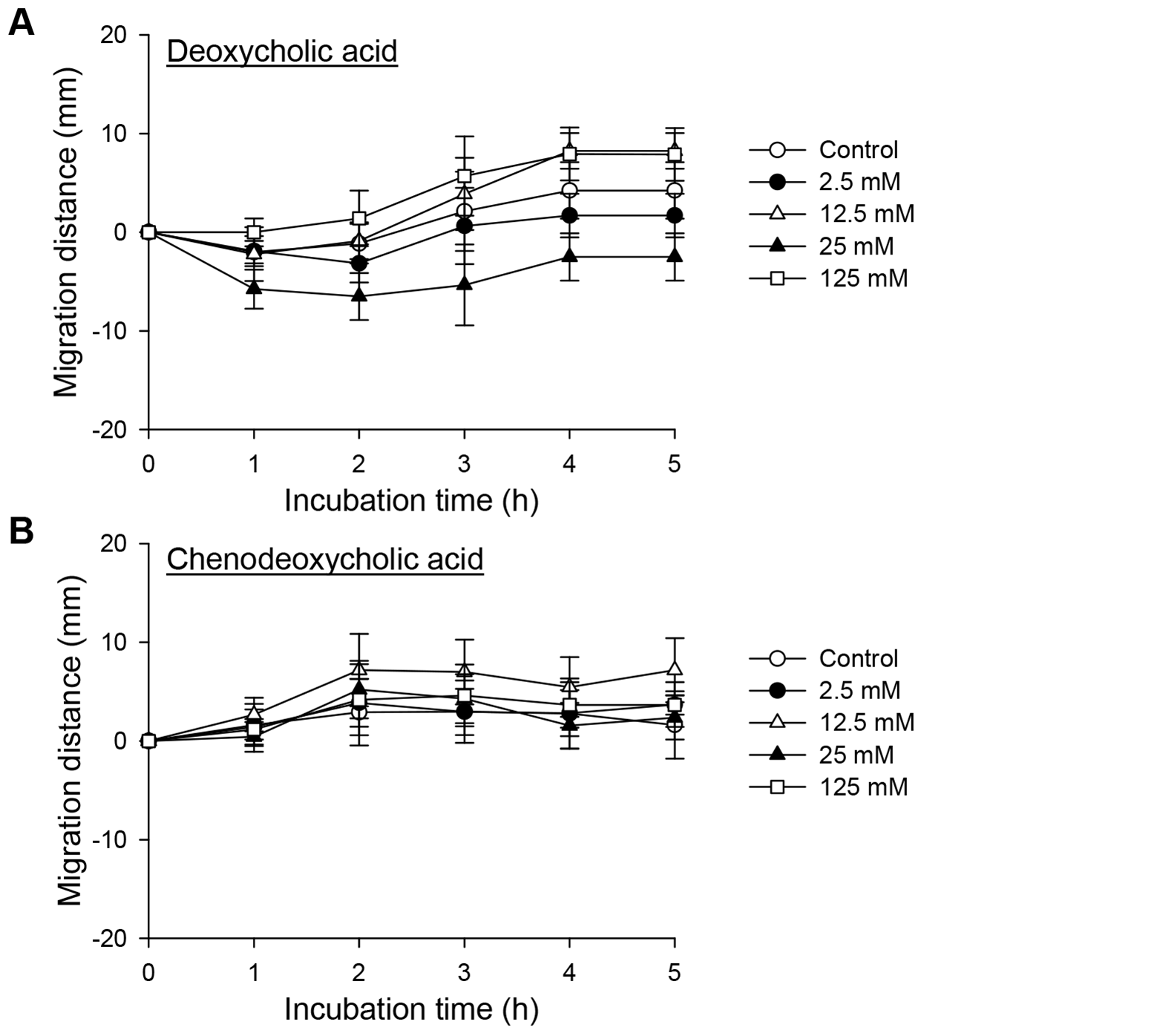

Supplement: S3 Fig — Chemotactic movement of CsNEJs was observed for 5 h in response to various concentrations of deoxycholic acid (A) and chenodeoxycholic acid (B). Each point represents mean ± standard error of mean. (TIF) [file pntd.0006818.s003.tif]
